# Provider, Caregiver, and Patient Experiences of an Integrated Care Program for Older Adults Designated as Alternate Level of Care: A Qualitative Case Study

**DOI:** 10.5334/ijic.7629

**Published:** 2025-03-24

**Authors:** Adora Chui, Kimia Sedig, Katie N. Dainty, On behalf of the NYCARES Design Team

**Affiliations:** 1 Research and Innovation, North York General Hospital, Toronto, Ontario, Canada; 2 North York Toronto Health Partners Ontario Health Team, Toronto, Ontario, Canada; 3 Institute of Health Policy, Management and Evaluation, University of Toronto, Toronto, Ontario, Canada

**Keywords:** integrated care, qualitative, case study, alternate level of care, homecare, caregiver

## Abstract

**Introduction:**

Following hospitalization, older adults with complex health and social care needs are often deemed to need an “alternate level of care” (ALC) where care needs are misaligned with resources. Coordinated networks can implement integrated care programs for this group in home settings. Understanding the experiences of providers, caregivers, and patients will inform ongoing implementation efforts.

**Methods:**

A qualitative case study was undertaken of North York Community Access to Resources Enabling Support (NYCARES), a novel integrated care program implemented during the COVID-19 pandemic. Data collection consisted of semi-structured interviews, document analysis, and observational field notes; data were thematically analyzed.

**Results:**

Thirty-six providers, caregivers, and patients were interviewed. Three themes were developed: 1) NYCARES as a lifeline; 2) Experiences tempered by expectations and connection; and 3) The role of integrated care.

**Discussion:**

The NYCARES program was seen as valuable, but implementation posed challenges for each participant group due to varying expectations and perceived degree of connection between patients, families, and providers.

**Conclusions:**

The local coordinated network successfully implemented the NYCARES program for ALC patients despite challenges in stakeholder connections. Similar programs should formally support caregivers and forefront multidirectional communication, particularly between providers in different implementation roles.

## Introduction

Complex patients, especially older adults, manage multiple interacting health and social conditions [1]. Often when they have been hospitalized, the plan for discharge can be complicated. They no longer require the intensity of hospital resources but may not be able to be safely discharged to their previous living situation, and frequently need more support that what is available in the community. In Ontario, these types of patients are designated as needing an Alternate Level of Care (ALC). Alternate Level of Care is challenging—for hospitals as this creates a bed flow bottleneck, and for patients who typically just want to go home and do not want to be in transitional limbo. The scale of the challenge with patients in this situation is costly, as more than 15% of hospital beds in the province of Ontario are occupied by ALC patients [2]. A common example is that those awaiting admission to a long-term care home typically have greater complex health and functional needs [3] but face a median wait of 90 days to be placed [2]. Caregivers are left to grapple with uncertainty regarding the discharge destination [4]. It is due to both the complexity of patient needs and the incomplete integration between health and social service sectors, that the transition is often challenging from hospital to home or another care setting [5,6].

The ALC challenge has been characterized as a manifestation of healthcare systems designed for acute conditions [7] and which is fragmented, negatively affecting care cost, quality, and outcomes [8]. There are opportunities to better manage complex and chronic conditions by deeply involving primary and community care through coordinated delivery networks and integrated care programs. Although integrated care programs have shown mixed results for the ALC population [9,10] and for older adults [11,12], recent evidence reviews have identified promising program elements including comprehensive assessment and case management [13,14] and care continuity and transitions [15]. These elements should be delivered in a setting that aligns with the person’s healthcare needs as judged by their goals, health status, and treatment plan [16].

A novel integrated care program for older adults was recently designed with these aspects in mind: North York Community Access to Resources Enabling Support (NYCARES) aims to transition individuals designated as ALC from the hospital to home with seamless, wraparound person-centred care to meet their complex needs [17]. Multiple health and social care services are available including medical oversight from a nurse practitioner, nursing visits, remote patient monitoring, occupational and physical therapy, homemaking, and personal support workers (PSWs). Services and equipment are deployed among acute, primary, and community organizations through care coordinators and a multidisciplinary team of providers. Upon consent to join the program, the patient and their family members/caregivers have an onboarding meeting with the provider team to co-create a care plan, including the planning of post-NYCARES destination (e.g., long-term care home, living at home with enhanced services) since it is a time-limited program of three months. A “care navigator” is the primary contact for each patient and their caregiver(s) for simplified access and support.

Although substantial generic knowledge about integrated care has been developed [18,19], we need to better understand the factors that drive behaviour, decision-making, collaboration, and implementation processes in integrated care networks [20]. Previously measured aspects of integrated care include access to healthcare services, effect on clinical outcomes, and cost-effectiveness [21,22,23]. Less is known about patient experience and provider experience compared with outcomes and cost in the Quadruple Aim framework [24]. While some exemplar approaches to integrated care for older adults [25,26] have been evaluated against important healthcare targets, the collective experiences of patients, caregivers, and providers have received less attention [e.g., 27]. Understanding the views, needs, and interactions of these “insiders” can illuminate relevant factors regarding implementation processes, impact, and sustainability. Early acquisition of patient and provider perspectives can also inform program decision-making before cost and outcomes data become available. The purpose of this study was to describe in depth how patients, caregivers, and providers experienced the implementation of the NYCARES integrated care program for ALC patients.

## Methods

### Methodology

This paper reports on the qualitative case study that was part of a larger process evaluation project investigating the impact of the context and mechanism of NYCARES on integrated care delivery (findings reported separately) for ALC patients. Qualitative research methods are well suited to contribute to a nuanced understanding of program implementation and how practice may deviate from design. A qualitative case study approach is useful for exploring and describing complex phenomena such that a deep understanding of an issue is produced from participant perspectives [28,29,30], and suited for health system research in that relationships, contexts, and programs can be examined as they evolve [31]. We chose this study design for its usefulness where the phenomenon and context are blurred [cf., 32], as is the case for NYCARES because it was designed and rapidly implemented during local health system transformation.

We utilized Merriam’s [29] pragmatic constructivist approach to case study research, focusing on NYCARES (particularistic) for a thick description of the program (descriptive) to yield an in-depth understanding of transitional care for the older adult ALC population (heuristic) [33]. As researchers, we were interpreting participant constructions of reality by the research process [29] and viewed participant language as indicative of their own realities, though we interpreted their words as part of the meaning-making process. We incorporated aspects of Stake’s [34] flexible design approach to allow for “progressive focusing” (p.22) as relevant directions became apparent (e.g., asking different probing questions during the interviews), but to remain rooted in the research objectives. Merriam’s and Stake’s approaches share concordant epistemological commitments, data collection techniques, and overlapping of data collection with analysis [31,33]. Three data collection methods were used in this study: observation, document analysis, and stakeholder interviews [29,34].

### Positionality

The research team was comprised of experienced qualitative researchers with expertise in implementation science (AC, KS, KND). At the time of the study, the first author was a postdoctoral researcher embedded within the NYCARES implementation team and tasked with conducting the process evaluation (but not involved with program design). The first author’s position was informed by her clinical background in geriatric occupational therapy which was valuable in communicating with patients and caregivers during the recruitment process and interviews. However, she presented herself as a researcher, not clinician, to the patients, caregivers, and providers. She also interacted with the implementation team as a researcher and was conscious of how analyzing provider interview data might affect her perception of team members (some team members were interviewed, though not by the first author). She engaged in reflexive journaling to cultivate self-awareness of any internal conflicts felt between what was observed during team meetings and what was said in interviews, and to process what and how to share emerging findings since they would influence the ongoing implementation.

### Study Context

The integrated care case reported here was defined as the NYCARES program. Located in a diverse, metropolitan area in Ontario, Canada, it was the flagship program for the area’s newly created coordinated network known as the local Ontario Health Team (OHT). Mandated by the provincial government, OHTs are a formalized partnering of health and social care organizations that are clinically and fiscally accountable for delivering more connected, coordinated care such that transitions between providers are made easier for patients and families [35]. The North York OHT was formed in December 2019 with 21 core partners. A working group of organizational leaders was convened to reimagine care for older adults with the most complex needs. Subsequently, NYCARES was launched in December 2020 during the COVID-19 pandemic, spanning two fiscal years under study: December 2020–March 2021 (NYCARES 1.0) and April 2021–March 2022 (NYCARES 2.0). More detail on the NYCARES program is provided in Appendix C.

### Participant Recruitment

We attempted to recruit as expansive a participant group as possible since the NYCARES program was multidisciplinary and intended to evolve in response to patient feedback. Patient and caregiver recruitment was done via convenience sampling with attempts to invite every NYCARES patient and their caregiver who had completed the program to participate. This was done through the care coordinators to ensure the first point of contact was through a care team member as required by research ethics.

A list of consenting names and contact information was then passed to researchers who continued the process of obtaining informed consent. Provider recruitment used a purposive sampling approach whereby the organizer of the NYCARES provider team supplied contacts of those who contributed to program design and/or implementation. Information letters were given to prospective participants prior to being contacted for an interview. Interviewers had no prior relationship with participants: AC interviewed caregivers and patients and KS interviewed providers. Participants were made aware of their interviewer’s research and/or NYCARES roles. Participants provided verbally recorded consent to participate. Ethics approval was granted by the North York General Hospital and University of Toronto research ethics boards.

### Data Collection

Observational data was collected via field notes at biweekly implementation team meetings to capture major implementation concerns, team dynamics, and contextual changes. Program documents (e.g., referral forms, financial invoices, funding applications, progress reports) were obtained via NYCARES’s online document repository and by requesting key materials. Documents were reviewed to understand the program rationale, design context, and implementation decisions and to be analyzed with other data sources.

In-depth, semi-structured interviews were conducted in English by phone or Zoom video platform with two groups: patients and caregivers (March–May 2022) and providers (October 2021–January 2022). The provider group included members from the administrative, clinical, and implementation teams. Different interview guides were used for each participant group. Four members of the North York OHT’s Patient and Caregiver Health Council advised on the interview guide for the patient and caregiver group, and we piloted the provider interview guide with two providers. No changes were deemed necessary from either group. Both interview guides consisted of open-ended questions with probes on experiences of NYCARES implementation and its key aspects (Appendices A & B). Interviews were audio-recorded and transcribed verbatim by an external transcription agency and checked for accuracy by the lead author. Interviewers also took field notes and engaged in reflexive journaling throughout the data collection periods.

### Data analysis

Data collection and analysis occurred concurrently. The research team judged that there was a fulsome range of participant experiences and that the data set was information rich after approximately 30 interviews. Interviews continued until all consented individuals were interviewed to be inclusive and engage everyone involved in the evaluation. Analysis was guided by Braun and Clarke’s reflexive thematic analysis method [36]. This began with coders engaging in several readings of interview transcripts to familiarize themselves with the full dataset. Conceptual notetaking was done across transcripts, observational data, and program documents (e.g., identification of key program design and implementation elements, timing of decisions, involvement of specific providers), and from there codes were delineated, formed into subthemes, and developed into themes. The first and last authors then met to discuss and refine the themes. This process happened iteratively and inductively, and reflexive notes of observations and documents were used to further inform our interpretation of interview data (e.g., supported theme development).

NVivo software was used to organize the analytic process of interview data. AC coded all interviews; KND coded three provider interviews and three caregiver interviews to support coherency of the coding approach and to augment richness of the understanding. We constantly revisited subthemes and themes, going between analytic levels (codes, subthemes, themes) to ensure an inductive, exploratory approach that was grounded in the data. Thematic maps were created for each group’s data set (providers vs. caregivers and patients) before being overlaid and refined by comparing consonance and dissonance of subthemes and themes between groups. We also shared a summary of findings to providers and to the consulting members of the Patient and Caregiver Health Council.

## Findings

Thirty-six individuals participated in 35 interviews: 18 providers, 17 caregivers (one spouse-child dyad), and one patient (Table 1 – Participant characteristics). Provider interviews lasted approximately 40 minutes (24–56 minutes), and patient and caregiver interviews lasted approximately 35 minutes (18–64 minutes).

**Table 1:** Participant characteristics.


DESCRIPTOR	n(%)

**Providers (n = 18)**	

Role	

Advisor (executive, patient council)	3 (17%)

Implementer	7 (39%)

Care coordinator	5 (28%)

Frontline service provider	3 (17%)

Program Developer (involved in initial design)	

Yes	11 (61%)

No	7 (39%)

Gender	

Female	16 (89%)

Male	2 (11%)

**Caregivers and Patient (n = 18)**	

Relationship to Patient	

Spouse or ex-spouse	3 (17%)

Sibling	2 (11%)

Child	12 (67%)

Patient	1 (6%)

Age	Average: 63.3 years (SD: 9.3)

	Median: 62.5 years

	Range: 49–82 years

Gender	

Female	11 (61%)

Male	7 (39%)

Main language used at home	

English	11 (61%)

Russian	4 (22%)

Polish	1 (6%)

Arabic	1 (6%)

Mandarin	1 (6%)

Fiscal year enrolled in NYCARES	

NYCARES 1.0 (2020–2021)	8 (47%)

NYCARES 2.0 (2021–2022)	9 (53%)


We spoke to providers from various NYCARES roles belonging to eight core OHT partner organizations within the coordinated network. Within provider roles, “advisors” were not involved in the operational aspects of NYCARES, but “implementers” were (i.e., team members involved in day-to-day operations of the program). “Care coordinators” coordinated client care and “frontline service providers” delivered patient care services. In the other participant group, most caregivers were children of the NYCARES patient. The average age of participants was 63.3 years (49–82 years). Unfortunately, only one patient was well enough to be recruited from this complex population.

Integrated analysis of observational field notes, program documents, and interview transcripts generated three main themes describing NYCARES stakeholder experiences: 1) NYCARES as a lifeline; 2) Experiences tempered by expectations and connection; and 3) The role of integrated care. Providers were observed as being very aware of overarching and external factors affecting health system dynamics; in contrast, the caregivers and patient group detailed different aspects that were more related to managing their daily lives. Each theme is presented below with illustrative quotes from the data.

### NYCARES as a lifeline

Participants in both groups described NYCARES as a lifeline for caregivers and patients, a solution to desperate, consuming care situations. Providers viewed NYCARES as an appealing service for complex patient needs allowing them to support patients and caregivers better than through existing care models. The NYCARES program required caregivers to strike a balance on decision-making for or with patients, but gave them relief when they had felt inundated by complex patient needs, along with wondering how they would manage daily care and finances:

“And so, when [NYCARES] was offered to me, it saved my life. It literally saved my life. Because I was so overwhelmed with how I was going to manage and cope and whatever. So, it gave me a real respite.” [C15]

This ALC period before receiving NYCARES was observed to be a stressful time with very few options, evidenced by how difficult it was for caregivers to recall specific details of joining NYCARES and their perception of the program as their only viable choice.

One of the documented rationales provided for the creation of the NYCARES program was for patients to be in their homes for as long as possible. Many caregivers stated their preference for their family member to be at home and that NYCARES was the only way for that to be an option; providers also remarked that caregivers believed that the patient would have a better quality of life in their own home. The single patient participant also expressed their happiness at being back in their home with culturally specific elements provided through NYCARES (e.g., meal service from a community-based organization arranged by the care coordinator):

“I feel it’s a lot better than in the hospital… I feel at home. I’ve got Chinese food. I’ve got people helping me to do all the stuff. It’s a big help.” [C11]

Caregivers described feeling less burdened by participating in NYCARES. Some families mentioned having a PSW always with the patient was the difference maker that allowed them to resume their jobs; others who did not have around-the-clock care desired more service hours for the same reason. Reflexive notes from the document analysis included the observation that though NYCARES was initially designed with 24-hour PSW care, it was only deemed appropriate on a case-by-case basis. There appeared to be a lack of clarity on the 24-hour service criteria which caused confusion. Observational meeting notes reflected discussions around the cost and logistical burden of staffing around-the-clock service when PSWs were disparately located.

Providers viewed NYCARES as appealing to families because of the opportunity for more personalized care and access to consistent service providers, which was particularly important during the pandemic. Most caregivers noted their psychological relief from receiving NYCARES supports because they felt they were not alone in figuring out the next steps. Moreover, NYCARES as a transitional care program enabled caregivers to come to terms with their new reality:

“It gave me a chance to adjust to the changes in our lives and what’s happening to him mentally, physically, socially, you know?” [C15]

Providers provided patients with coordinated, timely access to needed services that they felt was impossible to get outside the program. Providers also supported caregiver decision-making on the appropriate care settings post-NYCARES. However, the provider-caregiver-patient relationships were sometimes strained by factors such as attempting to balance different opinions on long-term home options. Ultimately, providers felt NYCARES was vitally important for supporting caregivers, articulating upstream effects possible through integrated care:

“If the caregiver is well, then the person living with dementia is well. And if that caregiver isn’t and isn’t well-supported and isn’t given the knowledge that they need, then everything kind of falls apart for both of them. And now you have two patients.” [P01]

### Experiences tempered by expectations and connection

The experience of both groups we talked to was impacted by personal expectations and the degree of connection. While many caregivers and the patient valued the quantity and diversity of NYCARES services, they experienced variations in service quality and expressed a need to feel more connected to the team. Caregiver experiences were tempered by their expectations of how much the program would simplify their lives and by the degree of connection they felt. These expectations were mostly influenced specifically by whether the PSWs met their expectations since PSWs interacted most frequently (usually daily) with families. Highly involved caregivers found it draining to explain care routines repeatedly to PSWs, confirm staffing schedules, and check that they were properly trained to work with older adults:

“I seemed to be the king person over everything that happened. Everything came through me. And so, I was constantly in demand for decisions left, right, and center.” [C10]

“The caregiver who has emotional concerns about the patient should not also be the one being the primary contact for all of the stuff.” [C12]

Program documentation shared with the research team did not show anticipation of this issue. Providers seemed unaware of this unintended strain on caregivers who felt like they became coordinators themselves.

Care coordinators had their own areas of strain, describing the most demanding period of setting up wraparound services from multiple OHT organizations. This initial period had the most documented requirements and was the most time-sensitive and pressure-filled because care coordinators needed to assess program eligibility, determine service requirements, and arrange in-home services within a couple days. The care navigator role, envisioned to be the patient and caregiver’s single point of contact, was intended as a mechanism of reducing caregiver stress. Unfortunately, the care navigator was also doing care coordination, which compounded the strain on this individual during the setup period and seemed to sap their capacity to be the key contact for families. In fact, one caregiver [C05] questioned the feasibility of routing every call to the care navigator (“She’s probably getting buried alive in way too many phone calls.”), but one provider lauded this unprecedented, seamless service:

“[The care navigator] would go in… to see how they were doing, see how their families were doing, make sure they had the care they needed. I mean that doesn’t exist in our system, where you have one person literally meet you at the hospital. Go to your house. Stay with you through, you know, transition to long-term care and beyond.” [P15]

Overall, caregivers recognized exceptional team members such as dedicated PSWs and caring nurses. Observations made from reflexive journaling point to how integral the direct service providers are to the patient and caregiver experience, even though the engine of the NYCARES program was envisioned as the behind-the-scenes coordination among multiple OHT organizations that enabled the provision of comprehensive, wraparound services. Further, the degree of caregiver connection with coordinators greatly affected the caregiver experience, because they felt more connected at the program’s start when communication was frequent. Nearing the end of services, however, some caregivers described being ill-prepared and stressed about what would happen next, especially given that post-program arrangements were communicated on relatively short notice.

Providers, in contrast, described having to figure out challenges throughout the process, which included dealing with frustrating operational inefficiencies. Their experiences were typically affected by an interplay between contextual factors (i.e., at healthcare system and program levels) and personal factors (e.g., commitment, roles and responsibilities). Most providers were “doing this program at the side of their desks,” [P16] meaning that they carved out time and energy from their existing responsibilities to be involved in NYCARES work. Providers motivated themselves by recalling the vision of the OHTs, that they “share the same vision of trying to coordinate care and providing more support for the clients.” [P04] They relied on provider-provider communication, trustful relationships, and a collective attitude of “figuring it out together.” [P08]

Benefits to this can-do approach were that providers felt a sense of ownership in organically developing structures and processes for NYCARES based on lessons learned along the way. Yet common challenges tended to stymie coordination efforts such as digital silos and the lack of shared integrated health record. Providers were further frustrated by unresolved issues. Conceptually, implementers and coordinators were uncertain about program goals and whether the program should be more accessible:

“There’s probably very similar patients out there that would not get anywhere close to that level of care or service… they wouldn’t have necessarily met the eligibility criteria.” [P06]

Operational inefficiencies were also raised by frontline service providers and care coordinators as problematic; their requests for change made to the implementation team often stalled so that they resorted to solving problems themselves. Field notes were frequently taken at implementation meetings on how similar issues when broached, like how the referral form needed updating, resulted in little action. Consequently, some frontline providers felt less connected to the overall implementation team:

“We don’t get time with them. We don’t know what they want. But we’re trying. So, the issues have been brought up.” [P16]

Likewise, implementers felt disconnected from their leadership group whom they viewed as having decision-making authority on program direction. Reflexive notes detailed angst for each group on delayed changes, particularly due to how busy each group was with day-to-day operations. Better communication and connection were observed within role categories (e.g., coordinator to coordinator) than between role categories (e.g., coordinator to implementer).

### The Role of Integrated Care

Despite implementation challenges, caregivers felt that NYCARES is a program that functions as a whole, and providers did feel like a cohesive team. Providers specifically appreciated how NYCARES was “filling in the gaps” [P04], meeting patient and caregiver needs in ways that other programs did not. They specifically highlighted the ability to share PSW coverage among OHT organizations and offer multisectoral services:

“This is really what we’re working towards. How do we create a system that is greater than the sum of its parts that really enables more compassion across our community?” [P09]

Providers were observed as grateful to be part of a high performing team that crossed traditional sectors of care, despite feeling disconnected to other provider groups. Although not perfect, individuals still articulated an overall sense of cohesiveness and enjoyed being part of a team with a sense of belonging, mutual respect, and group culture marked by:

“…a very strong similar work ethic among all of us. I would say quite – everyone is quite easily accessible, and responsible, and extremely efficient and effective.” [P08]

At the same time, a few advisors and implementers felt that the work was not truly collaborative but that they were working in parallel. Caregivers rarely detected any disconnection among providers, though they were unlikely to interact with those in advisor or implementer roles. Rather, many caregivers experienced the NYCARES program as flexible and responsive to their requests, particularly through the care navigator who “supplied us with all the information… looked after all the necessary arrangements.” [C07] However, one caregiver was highly critical of the healthcare system in general and upset that their family did not qualify for ongoing, in-home care. Most caregivers wished that their family member could have accessed this “best-kept secret” [C05] sooner:

“This program is outstanding. And when I talk to other people about it, you know, they were shocked that there is such a program and what was supplied.” [C15]

Caregivers suggested that more healthcare providers needed to learn about NYCARES and refer families before their situations devolved into crisis. Providers were similarly concerned and were trying to address this lack of public awareness.

Lastly, many providers had experienced recurring systemic problems over their careers, so they were glad therefore to contribute to an integrated care program offering innovative components, including virtual care. From program documentation, NYCARES had been designed with the expectation that virtual care would enhance quality of care and provider-patient communication. In experience, caregivers rarely mentioned virtual care or viewed it as an inappropriate option for patients with cognitive conditions. Instead of noticing only one service like PSW or nursing or occupational therapy, caregivers tended to be grateful for NYCARES as a package of integrated care:

“It’s all-encompassing… the suite of services that North York CARES has is very thoughtful and very worthwhile.” [C17]

## Discussion

This study describes how the implementation of NYCARES, an integrated care program focused on caring for ALC patients at home, was experienced by various stakeholder groups. The thematic analysis presented reflects the complex yet rewarding reality of program implementation within a newly formed integrated care network. Thirty-six participants were interviewed from three stakeholder groups—providers, a patient, and caregivers. All the groups we spoke with valued NYCARES and appreciated this integrated care approach as unique compared with other experiences they had had. However, each group had an incomplete picture of the implementation processes that influenced the behaviours of others, and divergent perspectives on which program aspects most impacted patient and caregiver experiences of transitional care.

The NYCARES program supplied some positive effects of integrated care previously identified in literature: perceived improved quality of care, increased satisfaction, and improved access to care [20]. The coordinated and cross-sectoral design of NYCARES appeared to immediately relieve urgent and multifaceted demands on caregivers, granting them time to adjust emotionally, psychologically, and financially. It may be prudent to prioritize integrated care approaches when suddenly increased care demands strain family structures beyond what can reasonably be absorbed. Ideally, programs like NYCARES should be widely publicized to healthcare professions and made standard at crucial points, such as after an unplanned hospitalization, before families reach a crisis mode. Case in point, the ALC designation is more likely to follow an unplanned hospitalization [37] when caregivers experience upheaval of routines and personal lives [38,39].

The integrated care ethos of being “an approach to strengthen people-centred health systems*…* designed around the multidimensional needs of the population and individual” [40] may be augmented by being increasingly *family*-centred—that is, to formally support caregivers within an integrated patient care approach. To this end, Kuluski and colleagues [4] have recommended that providers be trained to partner with caregivers directly during transitional periods, such as an ALC. The caregiver experience shared with us emphasized the importance of supportive relationships and having timely and sustained communication with providers—ingredients of interpersonal connection, as has been found to support health and well-being [41]. Greater partnership would likely enhance integrated care programs, but the onus must be on the providers so that caregivers can choose how and how much to be involved in the coordination aspects of these programs. This recommendation—explicitly providing caregivers with options on what caregiving aspects to be involved with and to what degree—seems rarely discussed in the caregiving literature [e.g., 42] which tend to focus on increasing caregiver involvement. Little instruction on building partnerships with families was present in program documentation; it would be prudent to augment the implementation process with more guidance on cultivating connection with caregivers throughout an integrated care program, particularly around inflection points like program start and end dates.

These insights can be further understood against Friedman and Tong’s [42] caregiver policy framework which identifies how caregivers can be integrated into health care teams. Of the six policy areas they discuss on reducing barriers to integration, three are part of the NYCARES design: identify and record information on family caregivers; incentivize providers to engage with family caregivers; and expand access to and funding for care coordinators to support caregivers and connect them to clinical information. Another area, “Develop, test, and improve caregiver access to technologies that foster caregiver-provider care integration and information-sharing,” was included as virtual care in NYCARES but the service was poorly utilized. Administrators and policymakers can bolster caregiver supports by improving the remaining two areas of the caregiver policy framework: invest in programs that provide supportive services for family caregivers; and implement training programs for providers and caregivers to facilitate effective communication.

Providers also experienced the growing pains encountered by introducing a novel integrated care program centred on multi-organizational collaboration and decision-making, factors also identified by Zonneveld et al. [20] as needing better understanding. Providers struggled with optimizing NYCARES processes due to concerns concordant with the integrated care literature, such as rearranging existing responsibilities and gaps in understanding the integrated care model [43]. For example, providers were hesitant regarding who the target population should be, perceptions of which have been found to influence implementation of integrated care programs [27]. Still, the overall provider experience affirmed that cross-sectoral organizations can join effectively around a shared vision and culture of team participation [15,44]. Perhaps reflecting provider team cohesiveness, caregivers perceived NYCARES to be a unified program, belying any operational turmoil among provider roles.

Providers further expressed their appreciation for being able to support families through cohesive teamwork that surmounted operational inefficiencies. This belief affirms that providers need to experience integrated care as superior to usual care to be contented and remain engaged [26]. Therefore, two factors which may help to overcome growing pains experienced by providers are to focus on team cohesion and to improve inter-group connection and program decision-making. Both factors require clear communication of workflows, expectations, supports, and roles and responsibilities required for integrated care implementation [43].

### Limitations

Interviews were conducted in English which may have been difficult for patient and caregivers for whom English is not their main language; however, there was ethno-cultural diversity with our sample. We were only able to interview one patient; however, this was not unexpected due to the multimorbid health situation for of the ALC patient population [32]. Although the patient’s view did not contradict other views, having more patient participation would have yielded richer descriptions of relevant program processes. Lastly, we did not interview PSWs because they did not participate in program design or coordination, but this design choice has limited our understanding of caregiver and patient interactions and perspectives of providers who were further removed from program design. Future studies should include contracted providers who were solely frontline providers of in-home care (i.e., PSWs, rehabilitation therapists).

## Conclusion

Both caregivers and providers recognized that NYCARES is filling a gap in the healthcare system, resulting in recognition of the integrated care approach made possible through a coordinated network of provider organizations. Our qualitative case study highlights a need to emphasize a family-centred (vs. patient-centred) approach to such integrated care innovations that intentionally supports caregivers of complex patients and that emphasizes communication and connection among stakeholder groups throughout implementation.

## Additional File

The additional file for this article can be found as follows:

10.5334/ijic.7629.s1 Appendices. Appendix A to C.
