# Supplementary material for: Provider, Caregiver, and Patient Experiences of an Integrated Care Program for Older Adults Designated as Alternate Level of Care: A Qualitative Case Study
Source: Int J Integr Care. 2025 Mar 24;25(1):12. doi: 10.5334/ijic.7629 (PMC11951966; doi:10.5334/ijic.7629)
Supplement: Appendices. — Appendix A to C. [file ijic-25-1-7629-s1.zip › ijic-25-1-7629-s1/671be1deea089.docx]

**Appendix A – Interview Guide (Patient and Caregiver)**

**Interview Questions *(probes in italics)*:**

1. Can you tell me a little about yourself? (relationship building)
   1. *Self-perceived health status?*
2. Please tell me about how you got connected with North York CARES. (key actors)
   1. *How did you learn about it?*
   2. *Who connected you?*
   3. *When did you meet your “Navigator”?*
   4. *How was your family member or caregiver involved?*
3. Why did you decide to join North York CARES? (decision-making during enrollment)
   1. *What attracted you to join North York CARES (e.g., instead of another program)?*
   2. *What were your expectations for the program when you started?*
4. What was the enrollment process like? (coordination, communication)
   1. *What instructions or information were you given at the beginning?*
   2. *Who was supporting your care planning? (e.g., patient, caregiver, interprofessional team)*
      1. *Did you have a good understanding of your shared care plan?*
      2. *Were you satisfied with your level of involvement in making your care plan? Why or why not?*
   3. *How did you feel when you were preparing to leave the hospital for home?*
      1. *Were your major concerns addressed? What were they? (e.g., transportation, PSW overnight care, caregiver stress)*
5. What services did you receive to be supported at home? (“menu of services” available)
   - 1. *Physician visits*
     2. *Nursing visits*
     3. *Personal Support Worker (PSW) care*
     4. *Community-based supports (e.g., meals, transportation)*
     5. *Other? (e.g., rehabilitation, social worker)*
6. *Did you receive virtual care? (if not aware, would you have benefitted from it?)*
   1. *What did you have set up for you (e.g., virtual visits, remote monitoring)?*
   2. *Did you find this service helpful? Why or why not?*
7. *Did the types of services you had change during your time in the program?*
8. What was your experience like of North York CARES? (patient-centred)
   1. *Who were the most important people you interacted with?*
      1. *Did you know who to contact if you needed to get in touch with the program?*
   2. *Were your priorities taken care of?*
   3. *Did you feel like your care team worked well as one team (was well coordinated)?*
   4. *In what ways did NYCARES help you?*
   5. *In what ways did NYCARES not work for you?*
9. Did you have what you needed to stay safely at home? (community needs)
   1. *Did you receive overnight care? (i.e., PSW care)*
   2. *Did you get equipment and/or supplies through North York CARES?*
   3. *Did you have to go back to the hospital at any point? Why or why not?*
10. What was your experience of finishing the program?
    1. *Did you feel prepared to transition out of North York CARES? Why or why not?*
    2. *How was your caregiver/family member involved in this transition?*
       1. *How did they feel about the program ending for you?*
11. Was there anything you would have wanted done differently?
    1. *How did North York CARES compare to your expectations at the beginning?*
    2. *Did you feel anything was missing from your care?*
    3. *What were the main elements that helped you (e.g., people, timing, services, coordination, communication)?*
    4. *Did you feel that any parts of the program were not needed?*
