# Supplementary material for: Provider, Caregiver, and Patient Experiences of an Integrated Care Program for Older Adults Designated as Alternate Level of Care: A Qualitative Case Study
Source: Int J Integr Care. 2025 Mar 24;25(1):12. doi: 10.5334/ijic.7629 (PMC11951966; doi:10.5334/ijic.7629)
Supplement: Appendices. — Appendix A to C. [file ijic-25-1-7629-s1.zip › ijic-25-1-7629-s1/671be1f4ca7e7.docx]

**APPENDIX B – Interview Guide (Provider)**

1. I’d love to start off the interview by learning a little more about you as a member of this program. Could you tell me a little bit about your role in this program?
   1. Can you describe your working environment in general terms?
   2. Tell me a little bit about how your branch or group is related to/works with the NYTHP as a whole?
   3. Can you tell me a little bit, what you know of the vision of the OHT?
      1. In your own words, how would you describe your relationship, or connection, to this vision?
         1. Tell me more; why or why not?
2. North York CARES involves multiple different program partners to meet the multi-dimensional needs of its target population. Can you tell me a little bit about what it’s like working with the other program partners? Describe to the best of your ability your relationship with the other partners in the OHT? North York CARES?
   1. What has been your experience with how roles and responsibilities are defined within your team? Between your team and other partner organizations?
   2. Can you describe your feelings when communicating ideas, initiatives, or suggestions among or across partner organizations?
3. Building on that, can you tell me a little bit about the atmosphere, or environment in your group? What do group members expect of each other? How do people usually behave? – another word for this could be culture
   1. What has your experience been with your group’s desire to improve its work?
      1. Any particular strategies or approaches that the group has taken, that you can think of?
   2. How do you think this does or does not align with the wider vision of the OHT?
   3. How do you feel about the resources at your disposal to act on improving this program’s work?
4. We know that the North York CARES program was developed to meet the very complex and multidimensional needs of a vulnerable population.
   1. How do you think the program is addressing the complexity of these needs?
      1. Tell me a little more about that. Are there particular areas that you feel the program is showing great potential? What about opportunities for improvement?
      2. How do you think North York CARES responds to the presence of those opportunities?
      3. How does this make you feel as a member of NYCARES? Outside of that role?
5. Having talked about [the program’s ability to address population needs], I wanted to talk a little bit about COVID-19. COVID has been a shock and an unprecedented ordeal for many this last year and a half. Do you think you could tell me a bit about what your experiences have been working as part of NYTHP during COVID?
   1. For example, in your experience how has the last year and a half impacted the program?
      1. COVID-19 as a phenomenon, specifically?
   2. How do you feel you have or have not been supported to continue the work you have been doing for those patients with complex needs under your care?
6. Can you tell me a bit more about the North York CARES program’s response to COVID-19? How has the program’s organization, daily routine, culture changed? How has it remained the same?
   1. Why? In what ways? Can you give some examples?
7. When I think of COVID-19, the word endurance comes to mind. Would you mind talking a bit about your thoughts on this program’s endurance?
   1. What does that mean to you? In your experience?
   2. Why?
8. How do you feel about the resources you have available to continue your work with the North York CARES program pre-COVID? Post-COVID?
9. Do you think there were any particular conditions in place that helped or did not help your organization’s response to COVID?
