# Supplementary material for: Provider, Caregiver, and Patient Experiences of an Integrated Care Program for Older Adults Designated as Alternate Level of Care: A Qualitative Case Study
Source: Int J Integr Care. 2025 Mar 24;25(1):12. doi: 10.5334/ijic.7629 (PMC11951966; doi:10.5334/ijic.7629)
Supplement: Appendices. — Appendix A to C. [file ijic-25-1-7629-s1.zip › ijic-25-1-7629-s1/671be20307a42.docx]

**Appendix C – Snapshot of the North York Community Access to Resources Enabling Support (NYCARES) Approach**

|  | **Introduction to NYCARES** | **Transition into and Setup of Services** | **Care Provision** | **Transition out of NYCARES** |
| --- | --- | --- | --- | --- |
| **Details of Care** | - Coordinator receives and reviews the referral - Patient & caregivers are involved in the goals of care discussion - Virtual care manager and a primary care provider are available if needed - Information is discussed with patient in segments to avoid overwhelming them - Transitions and backup plans are started | - Home visits set to discuss what is going on & how the client is feeling - Regular meetings (joint calls) to discuss care plans and appoint one key person to deliver information - Caregivers and patient support networks are engaged to identify unique patient needs, possibly through virtual visits - Cater services to how the client might perceive the care delivery - Caregivers engaged to understand resources and supports they need - Communication between primary care and home care - SMART goals set - Minimize handovers / provider changes - Strategies for PSW coverage | - Access to care and support through phone - Regular follow-up and progress reports on SMART goals - Feedback on what progress and improvements have been made - Medications check program | - Client and caregiver involved in the handoff and conversation - Both new and current care teams are involved in handoff conversations - New PSWs are present as the current PSWs completes the care |
| **People Involved** | - Pre-existing private care providers and home and community care providers - Referring provider(s) - Existing PSWs - NYCARES care navigator - Social worker - Regional home and community care coordinator - Pharmacist - Service coordination manager - NYCARES care coordinator - Primary care provider - Virtual care manager | - NYCARES care navigator - Community paramedics - Community home and community care coordinator - Peer navigator (program alum) - Social worker - Pharmacist - Medications Check program - Physicians who can provide after-hours care - Occupational therapist, physiotherapist - Personal support worker (PSW) supervisor | - Regular care team - Community paramedics - Group of physicians who can provide after-hours care | - Care team - NYCARES care navigator - Caregivers that the patient wants there (or that dynamics allow) - Organization assuming care (e.g., long-term care home) |
